# TIM3-mediated differentiation of IL-10-producing CD25^+^ B cells by expanded regulatory T cells

**DOI:** 10.1007/s00109-025-02606-0

**Published:** 2025-12-27

**Authors:** Rowa Y. Alhabbab, Daniela Mastronicola, Giovanna Lombardi, Cristiano Scottà

**Affiliations:** 1 Vaccines and Immunotherapy Unit, King Fahad Medical Research Center, King Abdulaziz University, Jeddah, Saudi Arabia; 2 Department of Medical Laboratory Sciences, Faculty of Applied Medical Sciences, King Abdulaziz University, Jeddah, Saudi Arabia; 3 Peter Gorer Department of Immunobiology, School of Immunology and Microbial Sciences, King’s College London, London, UK; 4 Department of Biosciences, Centre for Inflammation Research and Translational Medicine (CIRTM), College of Health, Medicine and Life Sciences, Brunel University of London, Heinz Wolff Building, Kingston Lane, Uxbridge, London, UB8 3PH UK

**Keywords:** Regulatory T cells, B cells, TIM3, CD25, IL-10

## Abstract

**Abstract:**

Cell-based immunotherapy utilizing regulatory T cells (Tregs) has recently advanced into clinical applications, demonstrating promising results in phase I/II trials to prevent transplant rejection and treat autoimmune diseases. We have completed a clinical trial in renal transplant patients in which the significant biological effect was the increase of B cells with a regulatory phenotype in the blood of kidney transplant patients. The mechanisms by which Tregs regulate B cells and the specific molecules involved in this process remained poorly understood. In this study, we employed an in vitro system of co-culture of peripherally purified B cells and expanded Tregs to show that Tregs can induce a population of memory B cells that express IL-10 and CD25. This subset of B cells has been previously identified as one of humans’ regulatory B cell populations. Notably, these expanded Tregs’ regulation of B cells was found to be independent of IL-10 and reliant on direct cell contact. We established that TIM3 expression by Tregs was crucial for the induction of IL-10-producing CD25^+^ memory B cells. Our findings suggest that TIM3 is a critical molecule for the induction of regulatory B cells by Tregs, indicating that TIM3 expression by adoptively transferred Tregs is vital in diseases where B cells play a pathogenic role.

**Key Messages:**

- Expanded Tregs induce IL-10+ CD25+ B cells.
- TIM3 expression on Tregs is crucial for IL-10+ B cell induction.
- Tregs require direct cell contact to regulate B cells.
- Blocking TIM3 reduces IL-10+ B cells but increases IFN-γ, TNF-α, IL-17.
- Tregs enhance regulatory B cell differentiation, promoting tolerance.

**Supplementary Information:**

The online version contains supplementary material available at 10.1007/s00109-025-02606-0.

## Introduction

Regulatory T cells (Tregs) are a small but crucial subset of CD4^+^ T cells that prevent autoimmune diseases and maintain immune homeostasis [1]. Numerous studies have demonstrated that impairments in the function or number of Tregs are associated with the development of various autoimmune disorders [2, 3]. Tregs exert their immunosuppressive effects by limiting the activation and proliferation of other immune cells through multiple mechanisms. These include the production of anti-inflammatory cytokines such as TGF-β, IL-35, and IL-10 and the expression of membrane-bound molecules like CD39, TIGIT, LAG-3, and CTLA-4. Additionally, Tregs can modulate the function of antigen-presenting cells through cell contact-dependent mechanisms, which alter the capacity of these cells for co-stimulation and antigen presentation. Their high expression of CD25 allows Tregs to sequester local IL-2, thereby limiting the expansion and function of effector T cells by depriving them of this critical growth factor [4].

Given their regulatory characteristics, Tregs have emerged as an attractive population for immunotherapy. In recent years, Tregs have been successfully isolated and expanded ex vivo in large numbers. Several phase I/II clinical trials have been conducted with promising results, some still ongoing [5], while others have been completed and shown some biological efficacy [6, 7]. Notably, our research has demonstrated that the infusion of polyclonal Tregs in kidney transplant patients leads to a dose-dependent increase in B cells with regulatory phenotype in the blood of treated individuals, suggesting that Tregs can influence B cell fate towards a more regulatory phenotype [8] and, more recently, an increase in another population of regulatory B cells (Bregs) has been seen in the first three renal transplant patients treated with Tregs in the TWO Study [9].

In humans, various B cell subsets have been identified that possess regulatory capacities through IL-10 production. These include transitional B cells (CD24^hi^CD38^hi^) [10], CD19^+^CD24^hi^CD27^+^ B10 cells [11], plasmablasts (CD27^inter^CD38^+^), TIM1^+^ Bregs [12], and CD25^+^ memory B cells [12, 13]. IL-10-producing B cells have been shown to modulate T cell responses by suppressing T helper 1 (Th1) and Th17 cells while promoting Treg induction [10, 14]. Furthermore, studies in autoimmune and transplant models indicate that the adoptive transfer of IL-10-producing B cells can improve disease outcomes [15, 16]. Despite these insights, the mechanisms by which Tregs regulate B cells and the specific molecules involved in their crosstalk remain poorly understood.

In this study, we demonstrate using an in vitro system that functionally enhanced ex vivo expanded Tregs (Exp-Tregs) are highly effective at inducing IL-10^+^ B cells, unlike freshly isolated Tregs (F-Tregs). We found that TIM3 expression on Exp-Tregs is essential for this effect. The induced IL-10^+^ B cells express CD25 and exhibit a memory phenotype (CD24^hi^CD38^−^). Our findings extend the understanding of the critical role of TIM3 in Treg function, particularly in Treg therapies applied in conditions where B cells contribute to pathogenic processes.

## Material and methods

### Human blood samples

All human blood samples were obtained from anonymous healthy donors with informed consent and full ethical authorization. Peripheral blood, collected as leukocyte-enriched blood cones, was supplied by the National Blood Service (NHS Blood and Transplantation, Tooting, London, UK). The Institutional Review Board of Guy’s Hospital granted this study’s ethical approval under reference number 09/H0707/86.

### Cell isolation and co-culture assays

Peripheral blood mononuclear cells (PBMCs) were isolated by lymphoprep (Stemcell Technologies, UK) density gradient centrifugation.

CD19^+^ B cells were enriched by negative selection via magnetic sorting (Miltenyi Biotec, UK). The purity of the B cells isolated with this protocol was always more than 95–98% by flow cytometry. For unstimulated B cell cultures maintained for 48 h, viability was ~49% (live cell gate), with about 81% of these events being CD19⁺ B cells.

To prepare activated γ-irradiated conventional CD4^+^ T cells (iTcells), CD4^+^ T cells were isolated using RosetteSep and incubated for 5 h with T Cell Activation Cocktail (without Brefeldin A), purchased from BioLegend (Cat# 423301). Each vial of this cocktail is supplied as a 500X stock and contains phorbol 12-myristate 13-acetate (PMA) at 40.5 µM (25 µg/mL) and ionomycin at 669.3 µM (500 µg/mL) in DMSO. When used at the recommended 1:500 dilution in culture, the final concentrations were PMA at 81 nM and ionomycin at 1.34 µm. After activation, the T cells were γ-irradiated and tested for CD40L expression by flow cytometry, and only preparations in which ≥96% of CD4⁺ T cells expressed CD40L were used in subsequent co-culture experiments. They were then cryopreserved at −80 °C and thawed immediately before use. The iTcells were utilized at a 1:4 ratio with B cells.

CD4^+^ T cells isolated by RosetteSep were also used for obtaining CD4^+^CD25^+^ Tregs by CD25 microbeads magnetic enrichment (Miltenyi), and FACS sorted using CytoFLEX SRT Cell Sorter (Beckman Coulter) and antibodies specific for CD4, CD25, CD127, and CD45RA. Tregs were then either used directly in our co-culture setting with the negatively sorted B cells or expanded by using anti-CD3/CD28 beads (Miltenyi) in the presence of 100nM rapamycin (LC-laboratories) and 1000 IU/ml recombinant human IL-2 in X-vivo15 medium (Lonza) supplemented with 5% human AB serum (Biosera), as previously published [17]. Exp-Tregs were collected and co-cultured with B cells (at a 1:1 ratio) in the presence of anti-CD3/CD28 beads for 48 h.

### Intracellular staining

PMA, ionomycin and brefeldin A were added for the last 4 h of co-cultures, and cytokines, including IFN-γ, TNF-α, IL-17 and IL-10, were measured by intracellular staining (ICC) acquired on a NovoCyte Flow Cytometer (Agilent Technologies).

### Trans-well system and antibody neutralization assay

Tregs and anti-CD3/CD28 beads were plated at the bottom of the well. The trans-well insert was placed on top, with B cells and iTcells (4:1 ratio). The plates were incubated for 48 h.

The anti-human TIM-3 monoclonal antibody (clone F38-2E2, BioLegend) used in this study has been shown to block TIM-3 binding to multiple ligands, including phosphatidylserine, CEACAM-1, and Galectin-9 [18, 19]. For blocking experiments, Tregs were pre-incubated with anti-TIM-3 monoclonal antibody (10 µg/ml) for 1 h at 37 °C. After incubation, cells were thoroughly washed in culture medium to remove unbound antibody. Subsequently, the anti-CD3/CD28 beads were added, and the cells were co-cultured with B cells and iTcells. The cells were then incubated for 48 h. PMA, ionomycin, and brefeldin A were added during the last 4 h of culture, and cytokines were measured by ICC using flow cytometry. This method was used to ensure that TIM-3 blockade was limited to Tregs and did not involve direct interaction of the antibody with B cells or iTcells.

### tSNE analysis and MFI normalization

For Fig. 2c, singlet live CD19⁺ B cells were gated and down-sampled to 5000 events per sample. Groups (non-stimulated, stimulated, and co-cultured with Tregs) were concatenated and analyzed together. t-SNE was run in FlowJo10 (iterations = 1000, perplexity = 30, learning rate = 200) based on CD24 and CD38 expression, IL-10 or CD25 were subsequently overlaid on the maps. Generally, singlet live cells were gated using Flowjo10, and additional gates were applied as requested. Singlet live cells of all samples were down-sampled to 5000 events, and each group (non-stimulated, stimulated and 1stimulated-Bcells:1Tregs) was concatenated into one file. tSNE was run on the concatenated files, and grouped data were gated. Cell clusters were identified and overlapped by the gated population on the tSNE map. For the MFI normalization, the MFI values were first obtained for each marker under all conditions. For visualization, data were normalized per marker, with the lowest MFI set to 0% and the highest set to 100%. This normalization was applied across all conditions for each marker.

### Statistical analysis

Comparisons between groups were performed using a T-test or two-way ANOVA and Tukey’s multiple comparisons as specified. Analyses were performed using GraphPad Prism software.

## Results

### Exp-Tregs induce IL-10-expressing B cells

Building on our prior evidence that following the adoptive transfer of Tregs, B cells with regulatory phenotype are increasing in renal transplant patients [20]. This study focused on understanding how expanded human Tregs impact B cell phenotype and function. To do so, we established an in vitro system in which human Tregs and B cells were activated by irradiated allogeneic T cells.

**Fig. 1:**
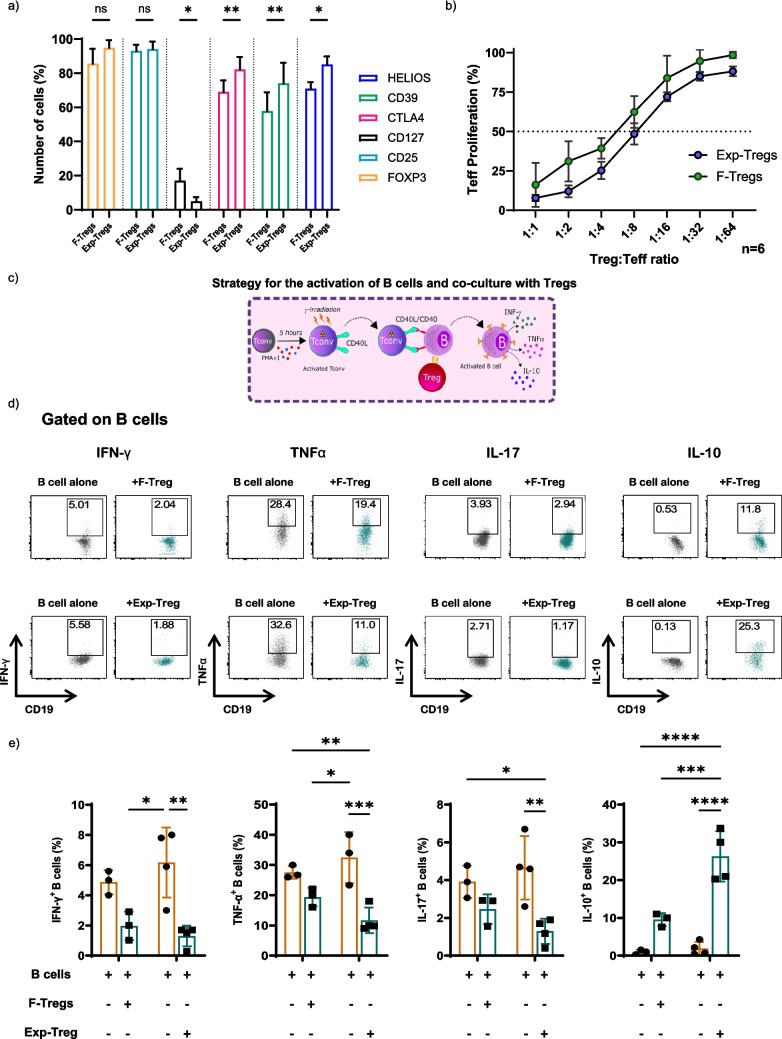
Exp-Tregs reduces pro-inflammatory cytokine production and induces IL-10 expression in B cells. Tregs were purified from the peripheral blood of healthy volunteers and ex vivo expanded with anti-CD3/CD28 beads in the presence of a combination of high doses of IL-2 and rapamycin. **a** Phenotypic characteristics of both F-Tregs and Exp-Tregs were assessed by flow cytometry after 5 days of culture. **b** F-Tregs and Exp-Tregs suppressive ability was evaluated by co-culture with varying ratios of CFSE-labelled conventional T cells (Teff) activated with anti-CD3/CD28 beads. **c** Schematic of the protocol used to activate B cells and co-culture with Tregs. **d** Representative FACS plots of CD19^+^ B cells producing IFN-g, TNFa, IL-17 and IL-10 following co-culture with F-Tregs and Exp-Tregs. **e** Summary data showing the production of IFN-γ, TNF-α and IL-10 in CD19^+^ B cells cultured alone or in the presence of F-Tregs and Exp-Tregs. Error bars represent mean ± SEM. *N* = 3 and *N* = 4 for F-Treg: B cell and Exp-Tregs: B cell co-culture, respectively. Statistical comparisons were performed using *t*-test or two-way ANOVA with Tukey’s multiple comparisons test, including direct comparisons between F-Tregs and Exp-Tregs. ns, not significant; *P < 0.05, **P < 0.005, ***P = 0.0005

Tregs were enriched from blood, expanded ex vivo using a well-established protocol in our laboratory, and used in previous clinical trials [21]. Briefly, Tregs were purified from the peripheral blood of healthy volunteers by density gradient separation of PBMCS, followed by magnetic bead separation of CD4^+^CD25^high^ T cells and fluorescence-activated cell sorting (FACS) to isolate F-Tregs. These Tregs were then stimulated with anti-CD3/CD28 beads (ratio 1:1, cell to bead) and cultured for 2 weeks in the presence of IL-2 (1000 IU/ml) and rapamycin (100 nM), as previously described [22, 23] to generate a highly pure and suppressive population of Exp-Tregs. At the end of the culture period, the purity of Exp-Tregs was assessed by flow cytometry using standard Treg markers and compared to F-Tregs. As shown in Fig. 1a, Exp-Tregs exhibited a slight increased expression of CD25, FOXP3, CTLA4, CD39 and HELIOS, while CD127 remained low compared to F-Tregs [24–26].

To evaluate their suppressive capacity, Treg were co-cultured at various ratios with CFSE-labelled conventional T cells (Teff) activated with anti-CD3/CD28 beads. In these experiments, Teff proliferation was assessed by flow cytometry through CFSE dilution, and Exp-Tregs suppressive function was compared to F-Tregs as previously described (Fig. 1b) [22, 23]. Exp-Tregs proved more suppressive than F-Tregs, further confirming their enhanced function.

Similarly, B cells (1×10⁷) were isolated from healthy donor PBMCs using CD19 magnetic beads (purity > 95%) and stimulated with iTcells that had been pre-treated for 5 h with PMA (81 nM) and ionomycin (1.34 μM) and confirmed to express high levels of CD40L (>96% positive by flow cytometry). These iTcells serve as a physiologically relevant source of CD40L and other co-stimulatory molecules, mimicking T cell help to B cells.

To investigate the cytokines produced by B cells after 48-h stimulation with only iTcells (baseline), we analyzed the percentage of B cells producing IFN-γ, TNF-α, IL-17, and IL-10, using intracellular staining (see the gating strategy in Supplementary Fig. 1a). As shown in Figs. 1c–e, activated B cells produced IFN-γ, TNF-α, and IL-17, while only a small fraction expressed IL-10. Negative controls are provided in Supplementary Fig. 1a.

At the same time, to test the effect of Tregs, activated B cells were co-cultured for 48 h at a 1:1 ratio with either F-Tregs or Exp-Tregs. Data in Fig. 1c–e showed that in the presence of both F-Tregs and Exp-Tregs, the percentages of B cells expressing IL-10 increased; however, the co-culture with Exp-Tregs resulted in even higher percentages of B cells producing this cytokine. Moreover, the percentages of IFN-γ, IL-17, and TNF-α were significantly reduced in B cells upon co-culture with F-Tregs and Exp-Tregs (Fig. 1d, e).

Cytokine expression was also analyzed in F-Tregs and Treg before and after the co-culture with activated B cells. Supplementary Fig. 1b-c shows that compared to F-Tregs, the percentage of Exp-Tregs expressing IFN-γ, TNF-α, and IL-17 was very low. In both F-Tregs and Exp-Tregs conditions, the percentage of TNF-α expressing cells was reduced following the co-culture with activated B cells. IL-10 production was only detectable in F-Tregs, and the co-culture with activated B cells reduced the percentage of cells producing this cytokine (Supplementary Figs. 1b-c).

### Exp-Tregs induce a regulatory phenotype in the memory B cell subset

**Fig. 2:**
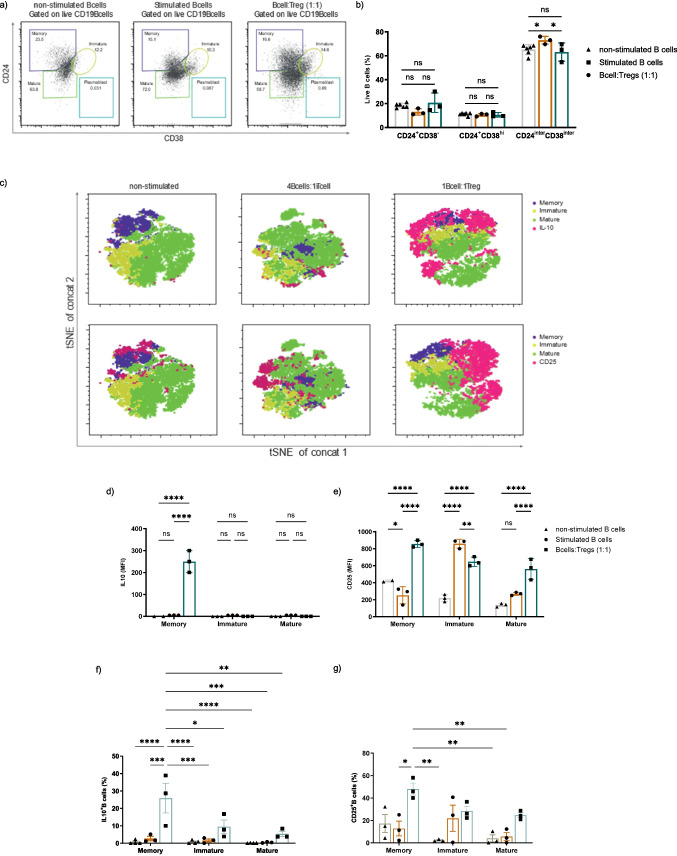
IL-10-expressing B cells have a memory phenotype. Analysis of B cell phenotype following 48-h co-culture with Exp-Tregs**.** **a** Representative FACS dot plots showing the CD19^+^ B cell subsets gating strategies. **b** summary data showing the frequencies of B cell subsets with and without Tregs. **c** t-SNE map showing B cell subset distribution based on CD24, CD38 and IL-10 expression (top row). The bottom row showing B cell subset distribution based on CD24, CD38 and CD25 expression on the same maps to illustrate the localization of IL-10⁺CD25⁺ cells within the CD24⁺CD38⁻ memory B cell subset. **d**–**g** Summary data showing IL-10 and CD25 MFI and percentages of IL-10⁺ and CD25⁺ cells among B cell subsets cultured with or without Exp-Tregs. Statistics were calculated by two-way ANOVA and Tukey’s multiple comparisons tests, ns—not significant, *P < 0.05, **P < 0.005, ***P = 0.0005, (*N* = 3)

Following the evidence that the co-culture of activated B cells with Exp-Tregs increased the percentages of IL-10-producing B cells, we sought to investigate the changes in the B cell subpopulations. B cells purified from the blood were stained with antibodies specific for CD24 and CD38 on CD19^+^ cells and analyzed by flow cytometry. Three different subpopulations of B cells were identified: CD24^+^CD38^−^ memory (CD24^+^CD38^−^), transitional (CD24^+^CD38^hi^) and mature (CD24^int^CD38^int^) B cells (Fig. 2a). The activation of B cells significantly increased the percentage of CD24^int^CD38^int^ mature subset, and this effect was inhibited by the presence of Exp-Tregs in the co-culture (Fig. 2b). The other B cell subpopulations, with or without the co-culture with Exp-Tregs, did not drastically change.

The phenotypic characteristics of B cells at the end of the co-culture were also analyzed by t-distributed stochastic neighbour embedding (tSNE); all data obtained from the co-culture conditions described above were combined to assess B cell subset distribution based on CD24 and CD38 expression along with the expression of IL-10 and CD25 (Fig. 2c). The analysis confirmed that Exp-Tregs mostly induced IL-10 production in B cells and that IL-10-producing B cells were clustered within the memory B cell subset (Fig. 2d). Further analysis of CD25 expression, a molecule previously associated with a regulatory phenotype in B cells producing high levels of IL-10 [6, 27], showed that co-culturing activated B cells with Exp-Tregs significantly increased this marker (Fig. 2e). Consistent with MFI analysis, frequency plots demonstrated a significant increase in IL-10⁺ and CD25⁺ cells specifically within the memory B cell subset (Fig. 2f, g and Supplementary Fig. 2a-b). Among the three defined B cell subpopulations, memory B cells expressed the highest level of IL-10 and CD25 compared to the other subsets (Fig. 2d, e). Notably, memory B cells were characterized as IgM^hi^, IgD^low^, and CD27^low^, which may indicate activation-related changes, selective survival, or in vitro differentiation within the memory compartment. Further studies are required to establish whether these cells represent precursors of CD27^hi^ memory B cells (Supplementary Fig. 2c) [7]. All these findings support the idea that the presence of Exp-Tregs induces the memory B cell subset to acquire a regulatory phenotype characterized by the high expression of CD25 and the production of IL-10.

### The expression of TIM3 on Exp-Tregs mediates the induction of tolerogenic B cells

**Fig. 3:**
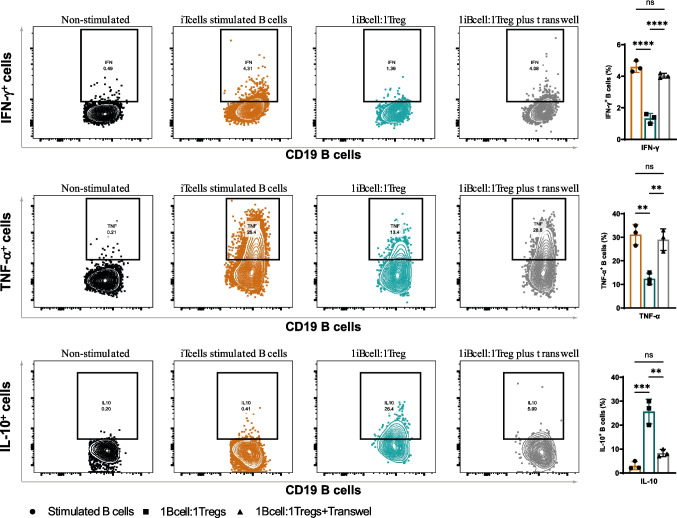
Exp-Tregs requires cell–cell contact to induce IL-10-expressing B cells. Exp-Tregs and B cells were cultured together on a transwell permeable support in a 24-well plate for 48 h. PMA, ionomycin, and brefeldin A were added to stimulate cytokine production for the last 4 h of culture. **a**, **b**, **c** Representative FACS plots of CD19^+^ B cell IFN-γ, TNF-α and IL-10 expression, and summary data showing IFN-γ, TNF-α and IL-10 expression by stimulated B cells alone and with Tregs in the presence and absence of trans-well insert. Statistics were calculated by two-way ANOVA and Tukey’s multiple comparisons tests, ns—not significant, *P < 0.05, **P < 0.005, ***P = 0.0005, (*N* = 3)

After observing that Exp-Tregs may influence the differentiation of a memory B cell subset with anti-inflammatory properties, we explored the mechanisms behind this effect. To test whether Exp-Tregs act through direct cell-to-cell contact, we used trans-well inserts to physically separate stimulated Exp-Tregs from stimulated B cells during a 48-h co-culture period. When Exp-Tregs were separated from B cells, the induction of IL-10^+^ B cells was prevented, and the reduction in IFN-γ and TNF-α B cells seen in co-cultures was also abolished (Fig. 3a–c). These findings indicate that Exp-Tregs required direct cell-to-cell contact to affect B cell properties.

**Fig. 4:**
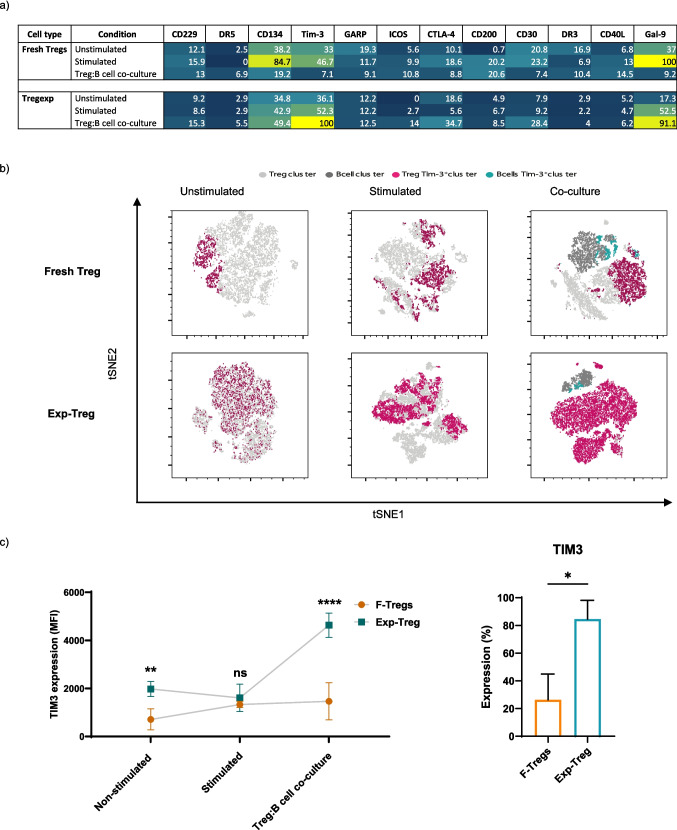
Exp-Tregs express high levels of TIM3. F-Tregs and Exp-Tregs phenotypic characteristics were analyzed by flow cytometry. F-Tregs and Exp-Tregs were left non-stimulated or stimulated with anti-CD3/CD28 beads or co-cultured with activated B cells (B cells:Tregs:iTcells at ratio 4:4:1) for 48 h. Tregs were stained for markers previously associated with their suppressive ability. **a**) Heatmap showing the normalized expression of the MFI data for each Treg-tested molecule. **b**) tSNE analysis of both F-Tregs and Exp-Tregs analyzed in the conditions described above. The manual gating defined Tregs and B cells using CD4, CD25, CD127 and CD19 expression in non-stimulated, stimulated Tregs and Tregs co-cultured with stimulated B cells. Analysis was performed on 5000 live singlet cells; samples were merged to generate a single tSNE map; TIM3 clusters were overlaid on tSNE maps to show TIM3 expression and distribution within Tregs and B cells. **c** Summary data comparing MFI and percentage of TIM3 in F-Tregs and Exp-Tregs Statistical analysis was performed using Two-way ANOVA and Tukey’s multiple comparisons tests, ns—not significant, *P < 0.05, **P < 0.005, ***P = 0.0005, (*N* ≥ 5)

To identify the mechanisms used by Exp-Tregs, we examined the expression of molecules associated with their regulatory functions and compared them to F-Tregs [28–37]. F-Tregs and Exp-Tregs were either left non-stimulated or activated with anti-CD3/CD28 beads, with or without activated B cells at a 1:1 ratio for 48 h. We evaluated the expression of CD134, TIM3, GARP, ICOS, CTLA-4, CD200, CD30, DR3, CD40L, and Galectin-9 (Gal-9) using flow cytometry. To visualize and confirm the most dominant molecule expressed by Exp-Tregs and at lower levels by F-Tregs under different culture conditions, we normalized the mean fluorescence intensity (MFI) data, setting the lowest value to 0% and the highest to 100% (Fig. 4a), and the mean of the raw actual MFI as well as the percentages of positive cells are also shown in Supplementary Fig. 3a and Supplementary Fig. 4.

Although Exp-Tregs expressed high levels of CD134, TIM3, CTLA-4, and Gal-9, we found that the co-culture of Exp-Tregs with B cells induced a very high expression of TIM3 and Gal-9 on Exp-Tregs. However, while similar levels of Gal-9 were also expressed on F-Tregs, the exceptionally high expression of TIM3 was restricted only on Exp-Tregs co-cultured with B cells (Fig. 4a).

The following analysis of the same samples using the tSNE algorithm (*n* = 5, 5000 events per sample) helped to visualize and identify the TIM3 distribution in the distinct cell clusters. Data in Fig. 4b shows that the co-culture of B cells with Exp-Tregs induced the whole population to express TIM3. Furthermore, Fig. 4c shows that when TIM3 expression was compared between the two preparations of Tregs, Exp-Tregs expressed considerably higher levels of TIM3 (MFI) compared to F-Tregs in all three different conditions (non-stimulated, stimulated and at 1:1 ratio with B cells). These results suggested that the expression of TIM3 by Exp-Tregs played a crucial role in the induction of IL-10^+^ memory B cells.

**Fig. 5:**
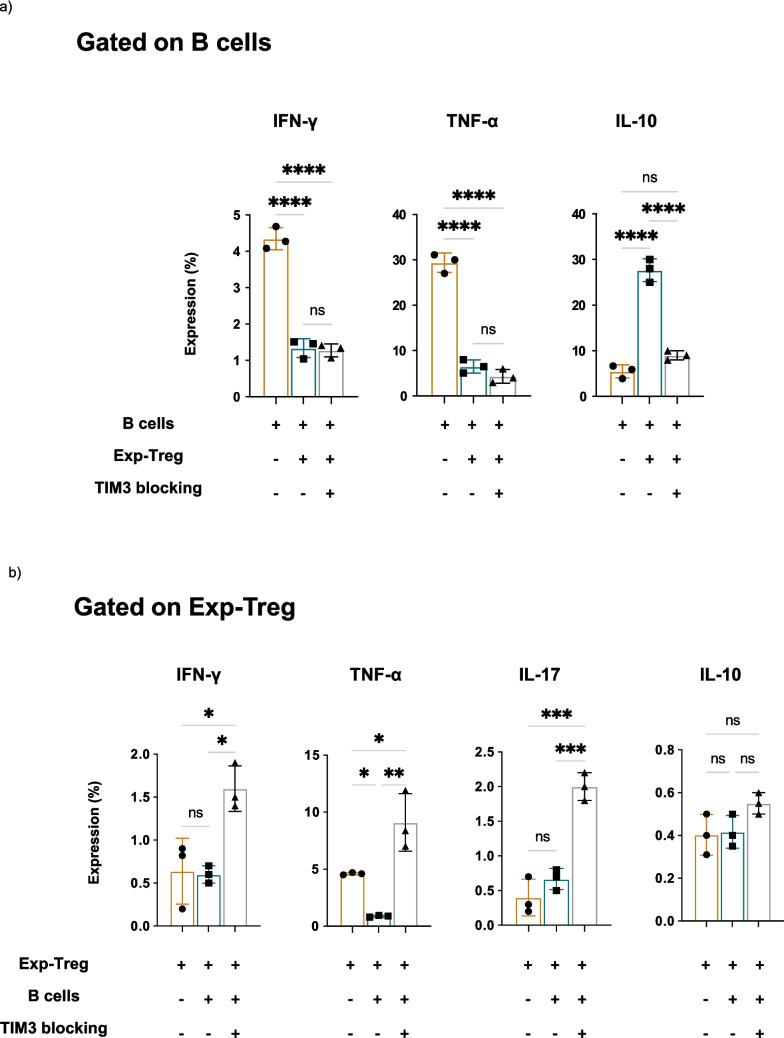
Exp-Tregs induces IL-10-producing B cells through TIM3. Intracellular staining of pro-inflammatory cytokines and IL-10 in B cells and Exp-Tregs. Exp-Tregs and B cells were cultured alone or together in the presence or absence of anti-TIM3 blocking antibody for 48 h. PMA, ionomycin, and brefeldin A were added to stimulate cytokine production for the last 4 h of culture. **a** Histograms show IFN-γ, TNF-α and IL-10 production (mean ± SEM) in B cells alone and with Tregs in the presence and absence of anti-TIM3 blocking antibodies. **b** Data show IFN-γ, TNF-α, IL-17 and IL-10 production (mean ± SEM) in Exp-Tregs alone and with B cells in the presence and absence of anti-TIM3 blocking antibodies. Statistics were calculated by two-way ANOVA and Tukey’s multiple comparisons tests, ns—not significant, *P < 0.05, **P < 0.005, ***P = 0.0005, (*N* = 3)

To confirm that TIM3 is involved in the crosstalk between Exp-Tregs and B cells, the two cell types were co-cultured in the presence of an anti-TIM3 blocking antibody. Additionally, a control experiment including non-stimulated B cells, iTcells stimulated B cells, and iTcells stimulated B cells with anti-TIM3 blocking antibodies alone was performed to ensure that the antibody does not exert a direct functional effect on B cells under these conditions (Supplementary Fig. 3b). Flow cytometry analysis showed a significant decrease in the percentages of IL-10^+^ B cells in the presence of anti-TIM3 (Fig. 5a), with only a minimal, statistically insignificant reduction in IL-10^+^B cells upon co-culture with F-Tregs (Supplementary Fig. 3c). However, blocking TIM3 did not affect the inhibition of IFN-γ and TNF-α in B cells (Fig. 5a). In the same culture conditions, the inhibition of TIM3 increased the percentages of IFN-γ, TNF-α, and IL-17-producing Exp-Tregs with no effect on IL-10 production (Fig. 5b). These results suggest that the crosstalk between Exp-Tregs and B cells is complex and requires multiple signals. While TIM3 engagement on Exp-Tregs plays a critical role in inducing IL-10 production in B cells, it is not necessary to inhibit proinflammatory cytokines such as IFN-γ and TNF-α. Conversely, blocking TIM3 signalling on Exp-Tregs stimulates the production of IFN-γ, TNF-α, and IL-17 by Exp-Tregs.

## Discussion

In this study, we utilized a novel in vitro co-culture system of human B cells and Exp-Tregs to demonstrate the potent ability of TIM3-expressing Tregs to induce memory IL-10^+^ CD25^+^ B cells. Importantly, our findings highlight that TIM3 expression is crucial for this effect.

Exp-Tregs offer significant advantages over F-Tregs, particularly in clinical applications. Exp-Tregs, similar to those utilized in clinical trials for liver and kidney transplantation (e.g., the ONE study and the THRIL study), exhibit enhanced functionality and stability. Notably, Exp-Tregs express high levels of TIM3, making them particularly effective at modulating T cell-mediated and B cell responses. This dual capability positions them as ideal candidates for therapeutic strategies promoting tolerance in transplant settings.

TIM3 is a molecule expressed on various immune cells, including B cells, T cells, monocytes, and dendritic cells. It plays a critical role in regulating immune responses and has been identified as a potential therapeutic target for several immune-related disorders, including cancer and sepsis. Recent literature has highlighted that tumors can exploit TIM3’s expression on tumor cells to evade immune detection [38]. Additionally, TIM3’s involvement in sepsis underscores its multifaceted role in immune regulation [39].

B cells are central to immune tolerance induction, with various subsets capable of downregulating inflammatory responses associated with autoimmunity and transplant rejection, primarily through IL-10 production [10, 15]. CD25^+^ B cells, initially recognized as a distinct subset, have been shown to differentiate upon stimulation via toll-like receptors [40]. These cells are now considered a regulatory B cell subset with memory characteristics that can enhance the Treg function [41]. Memory B cells, also known as B10 cells in humans, are particularly significant as IL-10 producers [42, 43]. While naïve B cells can convert CD4^+^CD25^−^ T cells into CD4^+^CD25^+^ Tregs [44, 45], our study uniquely demonstrates that memory B cells expressing IL-10 and CD25 can be induced by Tregs, specifically ex vivo expanded Tegs, emphasizing the critical role of TIM3 in this process.

The interaction between Exp-Tregs and B cells is complex and likely involves multiple communication mechanisms. The ligation of TIM3 with its ligand, galectin-9, is known to inhibit Th1 responses and promote peripheral tolerance [46–48]. TIM3 also plays a vital role in T cell exhaustion during chronic viral infections, where its inhibition enhances cytokine production specific to HCV and HIV [49, 50]. In cancer, blocking TIM3 signalling has been shown to improve the function of tumor-infiltrating lymphocytes [51]. Furthermore, reduced TIM3 expression is associated with the development of autoimmune diseases [52]. Interestingly, TIM-3 expression was comparable between F-Tregs and Exp-Tregs at baseline, yet diverged upon B cell co-culture, decreasing in F-Tregs but increasing in Exp-Tregs. This paradox highlights that TIM-3 regulation is highly context-dependent and may reflect distinct functional programs in these two subsets. Although TIM-3/Gal-9 interactions have been linked to inhibitory or apoptotic signaling, other studies suggest TIM-3 can also enhance Treg stability and suppressive activity [48, 53]. Thus, the increased TIM-3 expression on Exp-Tregs after B cell co-culture may represent an adaptive mechanism supporting their function rather than deletion. Further studies will be required to dissect these roles.

Importantly, we found that blocking TIM3 during the co-culture of Tregs and B cells explicitly influences the induction of IL-10 production in B cells without affecting the Treg-mediated suppression of pro-inflammatory cytokines such as IFN-γ and TNF-α. These findings suggest a complex interplay between Tregs and B cells that may involve additional molecular mechanisms.

Interestingly, the effect of TIM3 blockade on Tregs indicates that TIM3 also plays a role in regulating the expression of inflammatory cytokines in these cells. When TIM3 is inhibited, Tregs may begin to produce cytokines such as IFN-γ, TNF-α, and IL-17, which could compromise their regulatory function.

Our results are consistent with previous studies in both human and murine models, demonstrating that blocking TIM3 on stimulated conventional T cells significantly increases IFN-γ secretion [54, 55]. This underscores TIM’s essential role in T cell immunoregulation. Furthermore, while TIM3-expressing Tregs are more potent suppressors than TIM3-negative Tregs, they are also typically enriched in IL-10 [55, 56]. The discrepancies between these findings may stem from differences in the experimental models used, particularly the reliance on murine Tregs versus human cells.

Tregs are well-established for suppressing inflammation through various mechanisms, including the production of IL-10, TGF-β, and IL-35, as well as through direct cell contact [4]. Their role in alleviating the severity of diseases such as autoimmunity and transplantation has been well documented [4]. Consequently, Tregs have emerged as promising candidates for cell-based immunotherapy. They can be isolated and expanded ex vivo in large quantities, shifting the balance between effector T cells and Tregs, favouring the latter [57]. Our findings indicate that Exp-Tregs expressing TIM3 are crucial for inducing CD25^+^ memory IL-10^+^ B cells, contact-dependent, independent of IL-10. This suggests that TIM3^+^ Tregs enhance their suppressive capacity by promoting the differentiation of additional regulatory B cells, thereby creating a more tolerogenic environment.

Notably, although functional assays of IL-10⁺ B cells would provide further mechanistic insights, such experiments are significantly limited by the short-term survival and low frequency of these cells in vitro. IL-10 remains the most reliable surrogate marker of human Breg function, and its induction by Exp-Tregs offers a robust and biologically relevant readout of regulatory activity in our system.

In conclusion, our study demonstrates that Exp-Tregs, particularly those expressing TIM3, exhibit characteristics akin to exhaustion while maintaining robust suppressive functions. These Tregs effectively promote IL-10 expression in stimulated B cells with a memory phenotype. Leveraging TIM3^+^ Tregs could enhance their suppressive capacity and facilitate beneficial interactions with B cells, paving the way for innovative immune-based therapies.

## Supplementary Information

Below is the link to the electronic supplementary material.

ESM1 Supplementary Fig. 1. B cells induce small changes to Exp-Tregs. a) Gating strategy used to separate Exp-Tregs, iTcells and B cells in the coculture and representative FACS plots showing gating strategies for the isotypes controls used. b) representative FACS dot plots of IFN-g, TNFα, IL-17 and IL-10-producing F-Tregs and Exp-Tregs following co-culture with B cells. c) Summary data showing the production of IFN-γ, TNF-α and IL-10 expression in F-Tregs and Exp-Tregs co-cultured alone or with B cells. N = 3 and N = 4 for F-Treg: B cell and Exp-Tregs: B cell co-culture, respectively. Statistics were calculated by t-test, ns- not significant, *P < 0.05, **P < 0.005, ***P = 0.0005. **Supplementary Fig. 2. Phenotypic characteristics of B cells co-cultured with Exp-Tregs.** a-b) Representative FACS plots of the percentages of IL-10⁺ and CD25⁺ cells within each subset. c) Representative FACS plots and summary data of the expression (MFI and percentages) of IgM, IgD, and CD27 on non-stimulated B cells in the presence or absence of Exp-Tregs for 48 h. B cell subsets were identified by examining the expression of CD19, CD24, CD38, CD27, IgM, and IgD. d) Representative FACS plots and summary data of the percentages of IL-10⁺ B cells a control experiment including non-stimulated B cells, iTcells stimulated B cells, and iTcells stimulated B cells with anti-TIM3 blocking antibodies. e) summary data of the percentages of IL-10⁺ B cells upon stimulating B cells alone, with F-Treg with and without anti-TIM3. Data show mean ± SEM. Statistics were calculated by two-way ANOVA and Tukey’s multiple comparisons tests, ns- not significant, *P < 0.05, **P < 0.005, ***P = 0.0005, (n = 3). **Supplementary Fig. 3. MFI data of the molecules associated with Tregs’ regulatory functions and control for the anti-TIM3 blocking antibodies experiment.** a) Raw MFI values for molecules measured expressed on F-Tregs and Exp-Treg under three conditions: non-stimulated, stimulated alone, and co-cultured with B cells. These data correspond to the normalized values displayed in Fig. 4a and allow direct interpretation of the absolute magnitude of expression differences. b) Purified B cells were stimulated with iTcells for 48 h in the presence or absence of anti-TIM-3 blocking antibody (clone F38-2E2). c) F-Tregs were co-cultured with activated B cells at a 1:1 ratio with or without anti-TIM-3 for 48 h. Data show mean ± SEM. Statistics were calculated by two-way ANOVA and Tukey’s multiple comparisons tests, ns- not significant, *P < 0.05, **P < 0.005, ***P = 0.0005, (N ≥ 5). **Supplementary Fig. 4. Representative flow cytometry plots and summary expression data for selected co-inhibitory and co-stimulatory molecules on Tregs.** a) Representative flow cytometry plots are shown for the key molecules highlighted in the main text (TIM-3, CTLA-4, Galectin-9, and ICOS) on F-Tregs and Exp-Treg under the indicated culture conditions (non-stimulated, stimulated, and B-cell co-culture). Each plot illustrates the gating strategy used to define positive populations. Gating thresholds were determined using isotype control samples and applied uniformly across all conditions. b) Bar graphs summarize the percentages of positive cells values for all twelve molecules analyzed (CD229, DR5, CD134, TIM-3, GARP, ICOS, CTLA-4, CD200, CD30, DR3, CD40L, and Galectin-9) in both F-Tregs and Exp-Treg. Data show mean ± SEM. Statistics were calculated by two-way ANOVA and Tukey’s multiple comparisons tests, ns- not significant, *P < 0.05, **P < 0.005, ***P = 0.0005, (N ≥ 5) (PPTX 4.12 MB)

## Data Availability

The data supporting this study’s findings are available upon request from the corresponding authors.
