# Supplementary material for: TIM3-mediated differentiation of IL-10-producing CD25+ B cells by expanded regulatory T cells
Source: J Mol Med (Berl). 2025 Dec 27;104(1):18. doi: 10.1007/s00109-025-02606-0 (PMC12743028; doi:10.1007/s00109-025-02606-0)
Supplement: Supplementary file 1 — Supplementary Fig. 1. B cells induce small changes to Exp-Tregs. a) Gating strategy used to separate Exp-Tregs, iTcells and B cells in the coculture and representative FACS plots showing gating strategies for the isotypes controls used. b) representative FACS dot plots of IFN-g, TNFα, IL-17 and IL-10-producing F-Tregs and Exp-Tregs following co-culture with B cells. c) Summary data showing the production of IFN-γ, TNF-α and IL-10 expression in F-Tregs and Exp-Tregs co-cultured alone or with B cells. N = 3 and N = 4 for F-Treg: B cell and Exp-Tregs: B cell co-culture, respectively. Statistics were calculated by t-test, ns- not significant, *P < 0.05, **P < 0.005, ***P = 0.0005. Supplementary Fig. 2. Phenotypic characteristics of B cells co-cultured with Exp-Tregs. a-b) Representative FACS plots of the percentages of IL-10⁺ and CD25⁺ cells within each subset. c) Representative FACS plots and summary data of the expression (MFI and percentages) of IgM, IgD, and CD27 on non-stimulated B cells in the presence or absence of Exp-Tregs for 48 h. B cell subsets were identified by examining the expression of CD19, CD24, CD38, CD27, IgM, and IgD. d) Representative FACS plots and summary data of the percentages of IL-10⁺ B cells a control experiment including non-stimulated B cells, iTcells stimulated B cells, and iTcells stimulated B cells with anti-TIM3 blocking antibodies. e) summary data of the percentages of IL-10⁺ B cells upon stimulating B cells alone, with F-Treg with and without anti-TIM3. Data show mean ± SEM. Statistics were calculated by two-way ANOVA and Tukey’s multiple comparisons tests, ns- not significant, *P < 0.05, **P < 0.005, ***P = 0.0005, (n = 3). Supplementary Fig. 3. MFI data of the molecules associated with Tregs’ regulatory functions and control for the anti-TIM3 blocking antibodies experiment. a) Raw MFI values for molecules measured expressed on F-Tregs and Exp-Treg under three conditions: non-stimulated, stimulated alone, and co-cultured with B cel [file 109_2025_2606_MOESM1_ESM.pptx]

## Slide 1
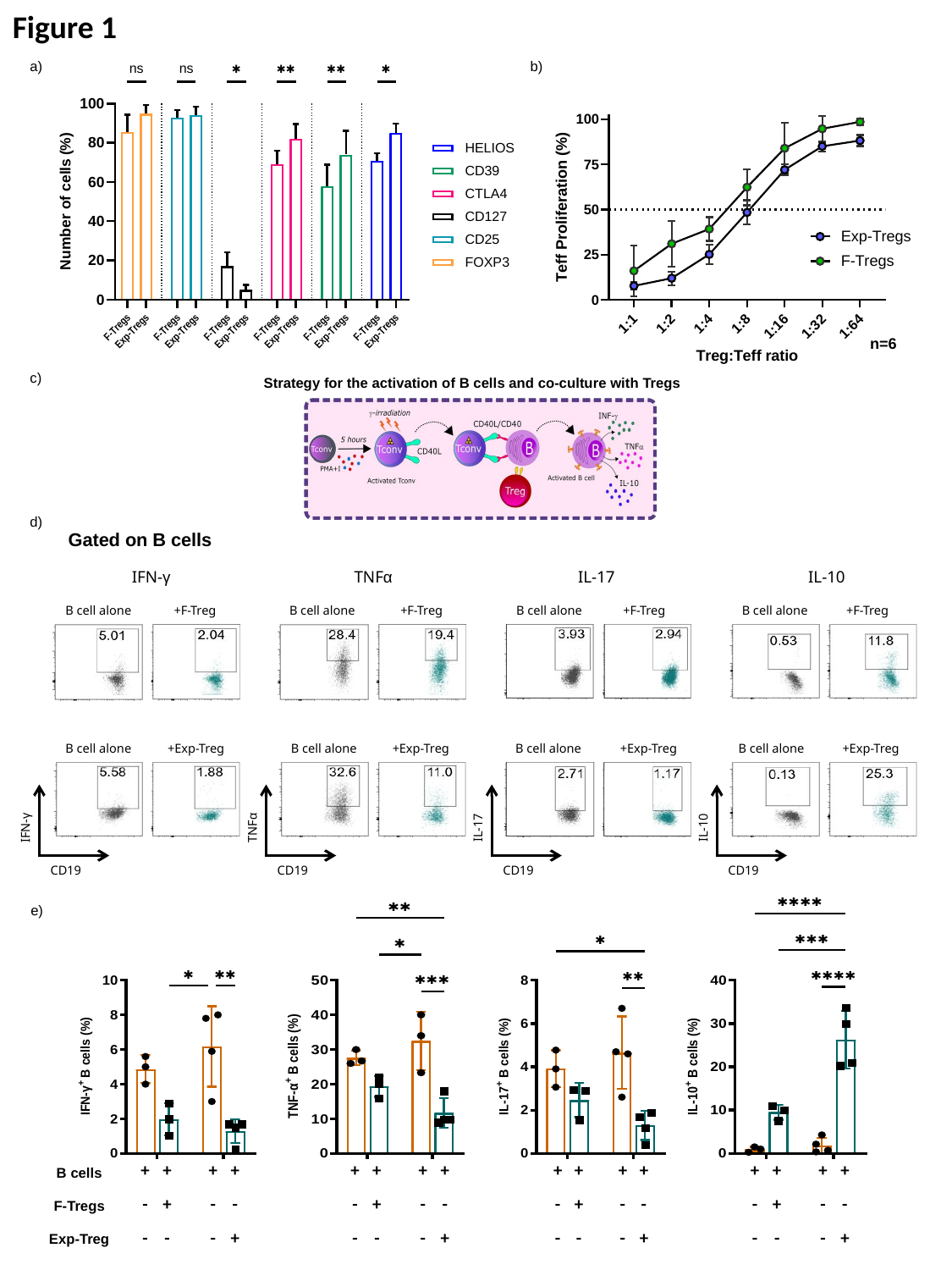

Figure 1
a)
b)
c)
Strategy for the activation of B cells and co-culture with Tregs
d)
Gated on B cells
IFN-γ
TNFα
IL-17
IL-10
B cell alone
+F-Treg
B cell alone
+F-Treg
B cell alone
+F-Treg
B cell alone
+F-Treg
B cell alone
+Exp-Treg
B cell alone
+Exp-Treg
B cell alone
+Exp-Treg
B cell alone
+Exp-Treg
IFN-γ
CD19
TNFα
CD19
IL-17
CD19
IL-10
CD19
e)
| + | + | | + | + |
| --- | --- | --- | --- | --- |
| - | + | | - | - |
| - | - | | - | + |
| + | + | | + | + |
| --- | --- | --- | --- | --- |
| - | + | | - | - |
| - | - | | - | + |
| + | + | | + | + |
| --- | --- | --- | --- | --- |
| - | + | | - | - |
| - | - | | - | + |
| + | + | | + | + |
| --- | --- | --- | --- | --- |
| - | + | | - | - |
| - | - | | - | + |
| B cells |
| --- |
| F-Tregs |
| Exp-Treg |

## Slide 2
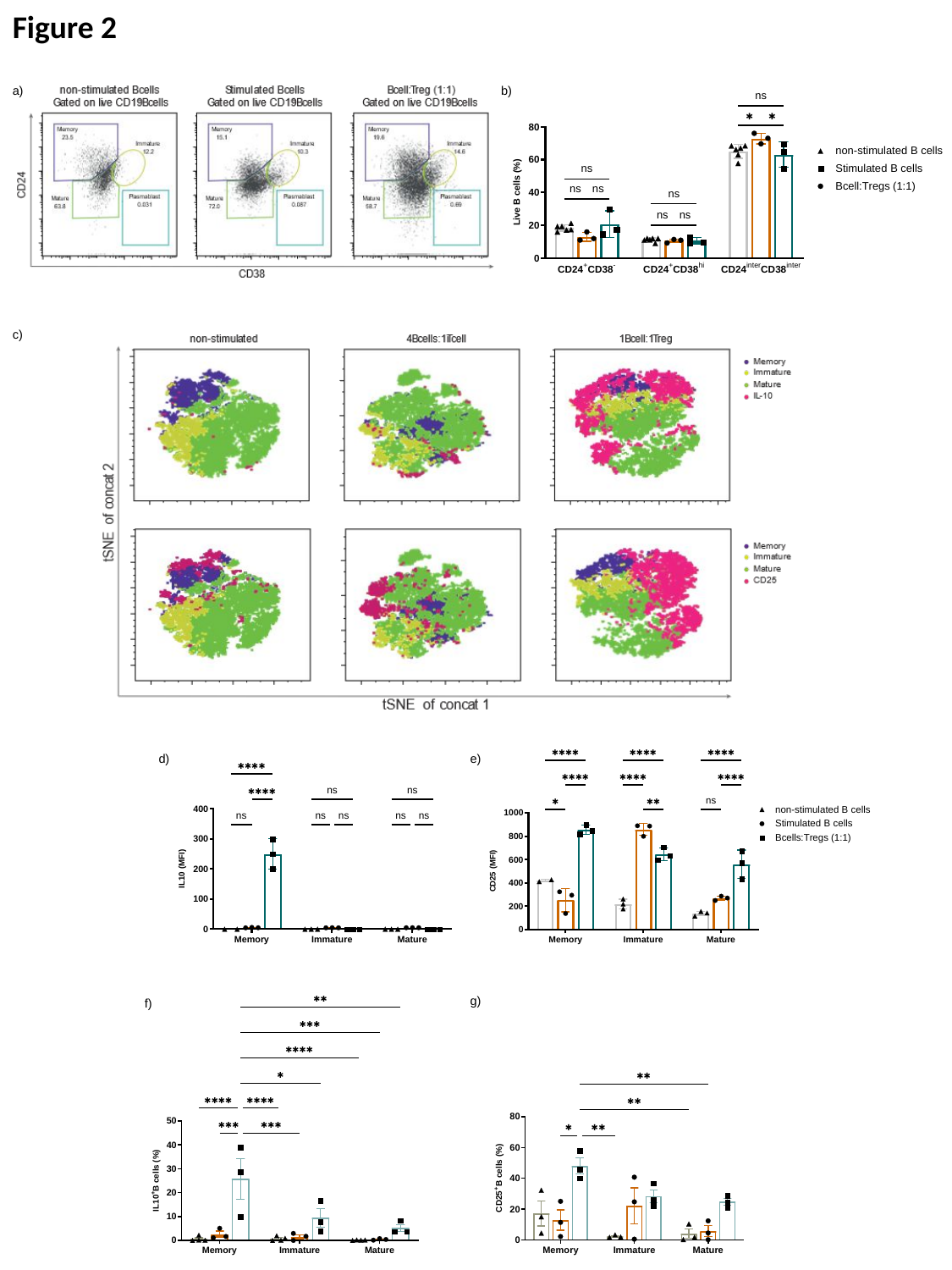

Figure 2
b)
a)
c)
d)
e)
g)
f)

## Slide 3
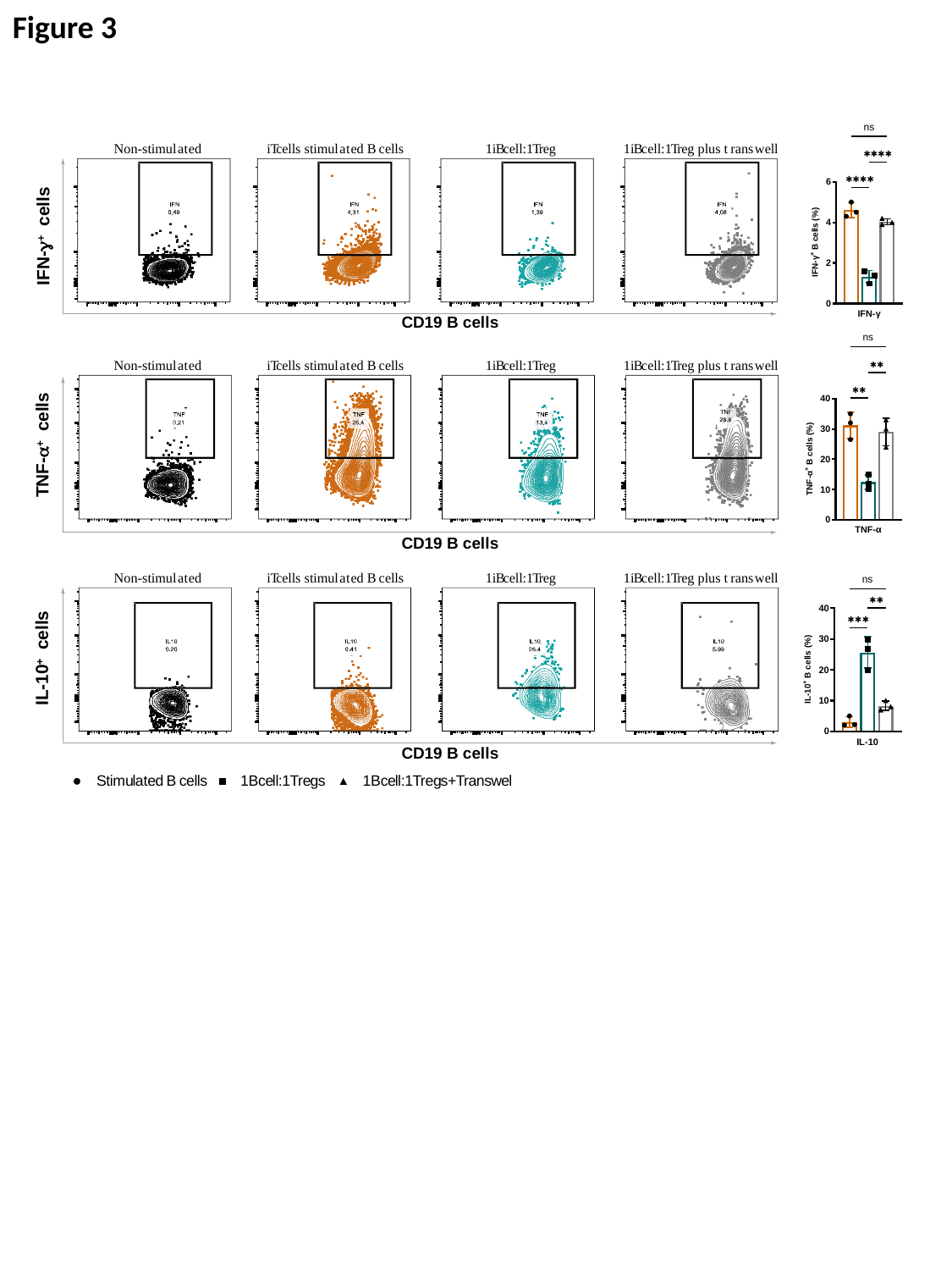

Figure 3
IFN-g+ cells
TNF-a+ cells
IL-10+ cells

## Slide 4
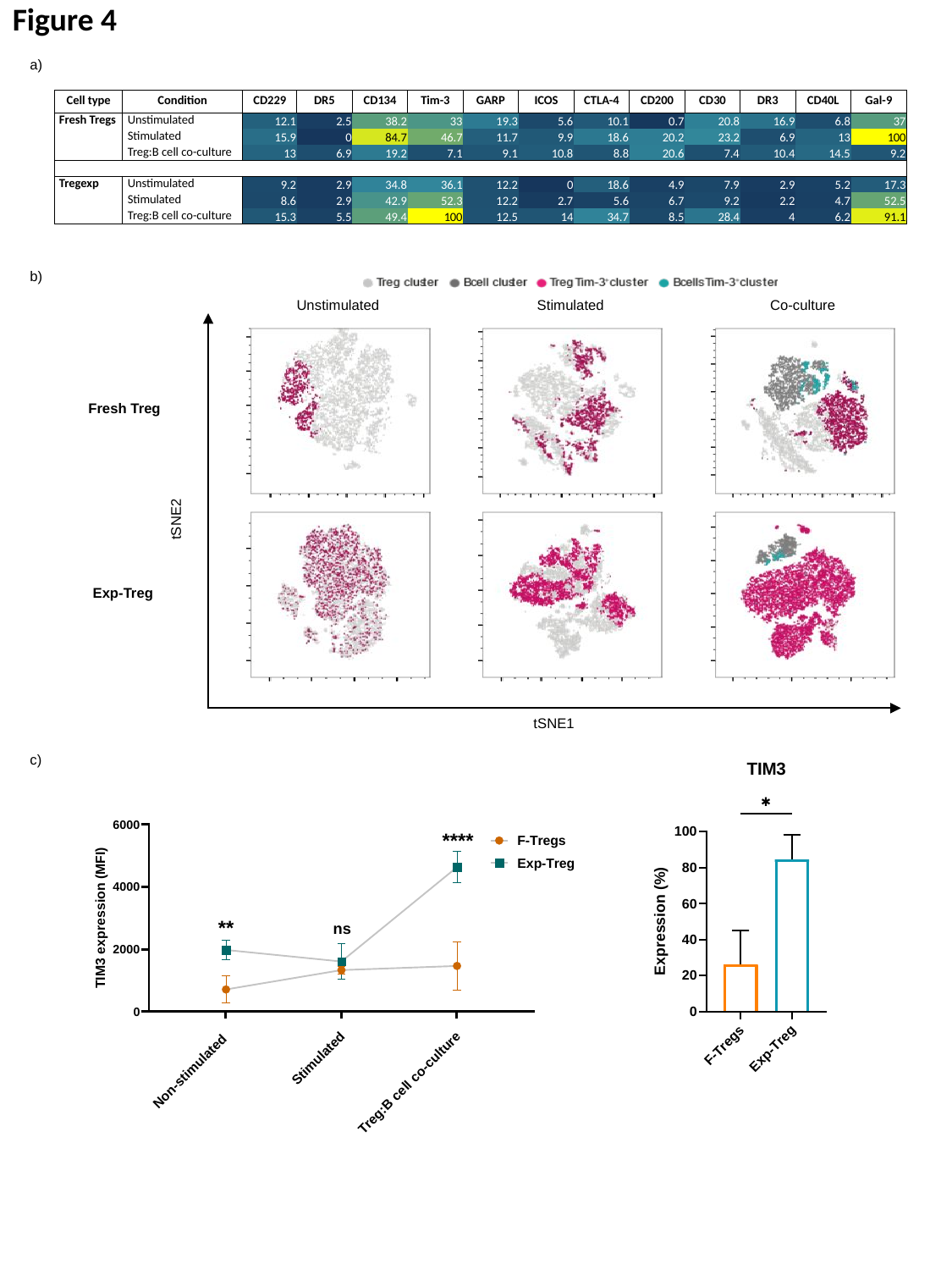

Figure 4
a)
| Cell type | Condition | CD229 | DR5 | CD134 | Tim-3 | GARP | ICOS | CTLA-4 | CD200 | CD30 | DR3 | CD40L | Gal-9 |
| --- | --- | --- | --- | --- | --- | --- | --- | --- | --- | --- | --- | --- | --- |
| Fresh Tregs | Unstimulated | 12.1 | 2.5 | 38.2 | 33 | 19.3 | 5.6 | 10.1 | 0.7 | 20.8 | 16.9 | 6.8 | 37 |
| | Stimulated | 15.9 | 0 | 84.7 | 46.7 | 11.7 | 9.9 | 18.6 | 20.2 | 23.2 | 6.9 | 13 | 100 |
| | Treg:B cell co-culture | 13 | 6.9 | 19.2 | 7.1 | 9.1 | 10.8 | 8.8 | 20.6 | 7.4 | 10.4 | 14.5 | 9.2 |
| | | | | | | | | | | | | | |
| Tregexp | Unstimulated | 9.2 | 2.9 | 34.8 | 36.1 | 12.2 | 0 | 18.6 | 4.9 | 7.9 | 2.9 | 5.2 | 17.3 |
| | Stimulated | 8.6 | 2.9 | 42.9 | 52.3 | 12.2 | 2.7 | 5.6 | 6.7 | 9.2 | 2.2 | 4.7 | 52.5 |
| | Treg:B cell co-culture | 15.3 | 5.5 | 49.4 | 100 | 12.5 | 14 | 34.7 | 8.5 | 28.4 | 4 | 6.2 | 91.1 |
b)
Unstimulated
Stimulated
Co-culture
Fresh Treg
tSNE2
Exp-Treg
tSNE1
c)

## Slide 5
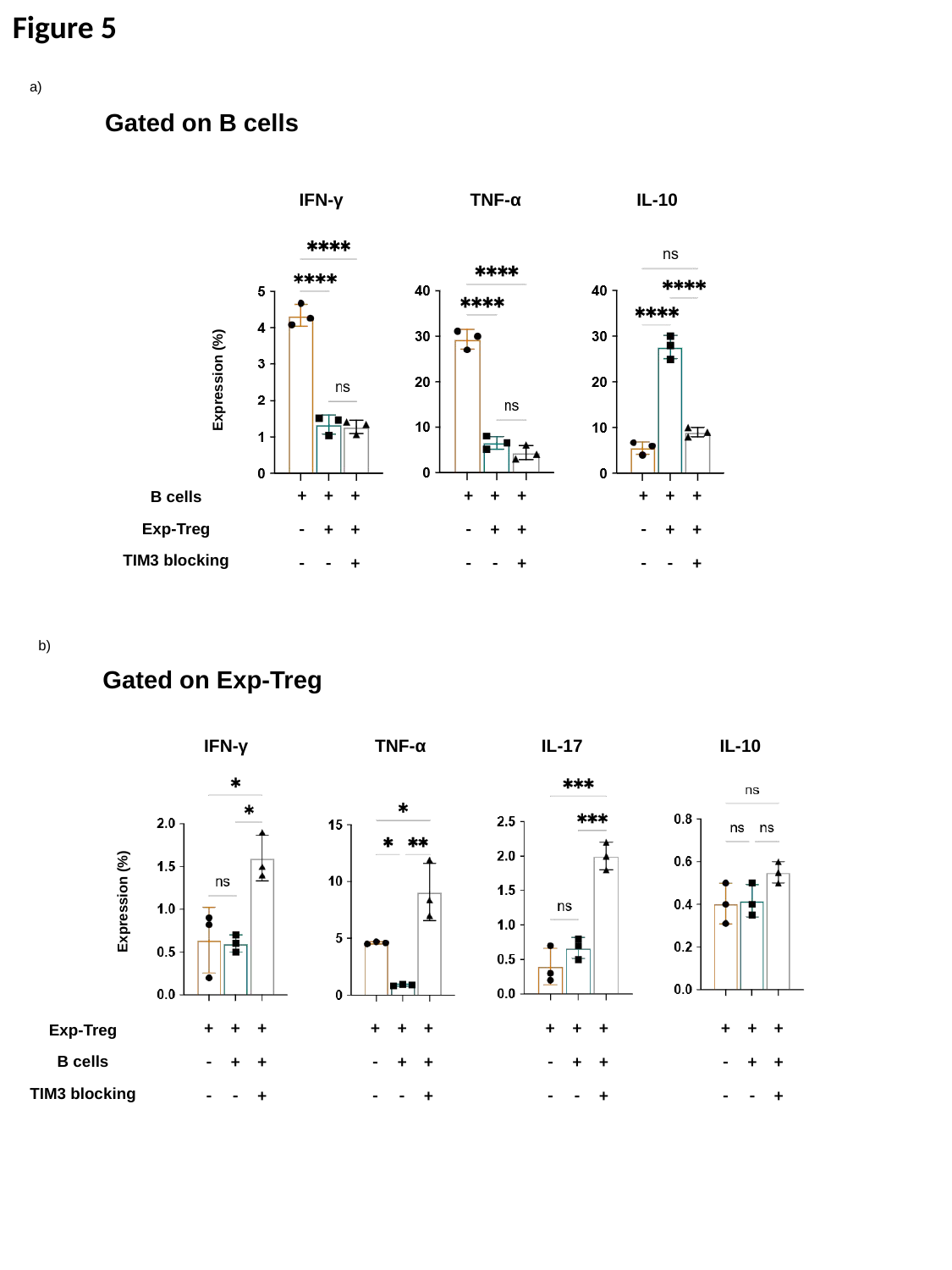

Figure 5
a)
Gated on B cells
IFN-γ
TNF-α
IL-10
Expression (%)
| B cells |
| --- |
| Exp-Treg |
| TIM3 blocking |
| + | + | + |
| --- | --- | --- |
| - | + | + |
| - | - | + |
| + | + | + |
| --- | --- | --- |
| - | + | + |
| - | - | + |
| + | + | + |
| --- | --- | --- |
| - | + | + |
| - | - | + |
b)
Gated on Exp-Treg
IFN-γ
TNF-α
IL-17
IL-10
Expression (%)
| Exp-Treg |
| --- |
| B cells |
| TIM3 blocking |
| + | + | + |
| --- | --- | --- |
| - | + | + |
| - | - | + |
| + | + | + |
| --- | --- | --- |
| - | + | + |
| - | - | + |
| + | + | + |
| --- | --- | --- |
| - | + | + |
| - | - | + |
| + | + | + |
| --- | --- | --- |
| - | + | + |
| - | - | + |

## Slide 6
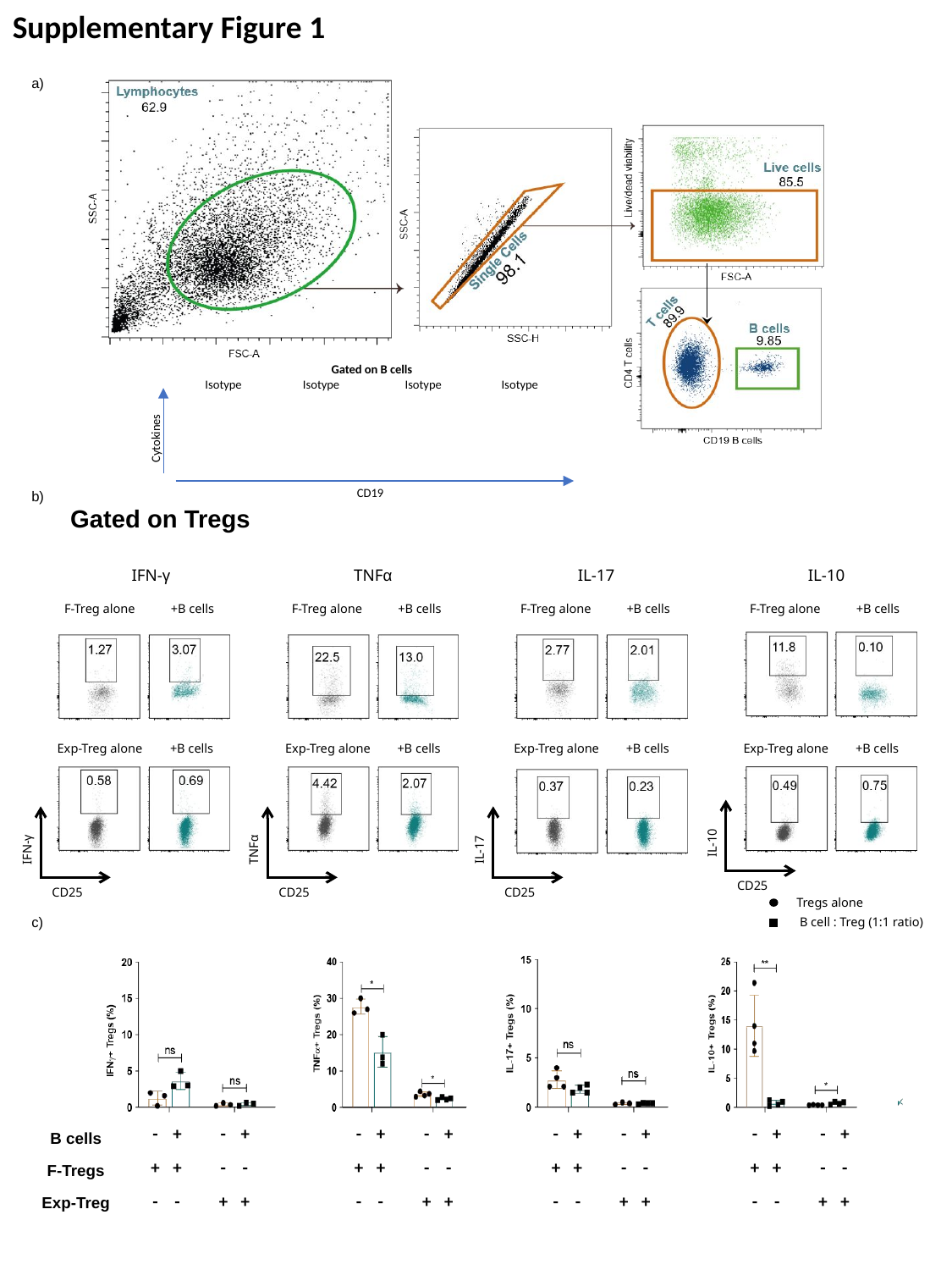

Supplementary Figure 1
a)
Gated on B cells
Isotype
Isotype
Isotype
Isotype
Cytokines
CD19
b)
Gated on Tregs
IFN-γ
TNFα
IL-17
IL-10
F-Treg alone
+B cells
F-Treg alone
+B cells
F-Treg alone
+B cells
F-Treg alone
+B cells
Exp-Treg alone
+B cells
Exp-Treg alone
+B cells
Exp-Treg alone
+B cells
Exp-Treg alone
+B cells
IL-10
CD25
IFN-γ
CD25
TNFα
CD25
IL-17
CD25
Tregs alone
B cell : Treg (1:1 ratio)
c)
| - | + | | - | + |
| --- | --- | --- | --- | --- |
| + | + | | - | - |
| - | - | | + | + |
| - | + | | - | + |
| --- | --- | --- | --- | --- |
| + | + | | - | - |
| - | - | | + | + |
| - | + | | - | + |
| --- | --- | --- | --- | --- |
| + | + | | - | - |
| - | - | | + | + |
| - | + | | - | + |
| --- | --- | --- | --- | --- |
| + | + | | - | - |
| - | - | | + | + |
| B cells |
| --- |
| F-Tregs |
| Exp-Treg |

## Slide 7
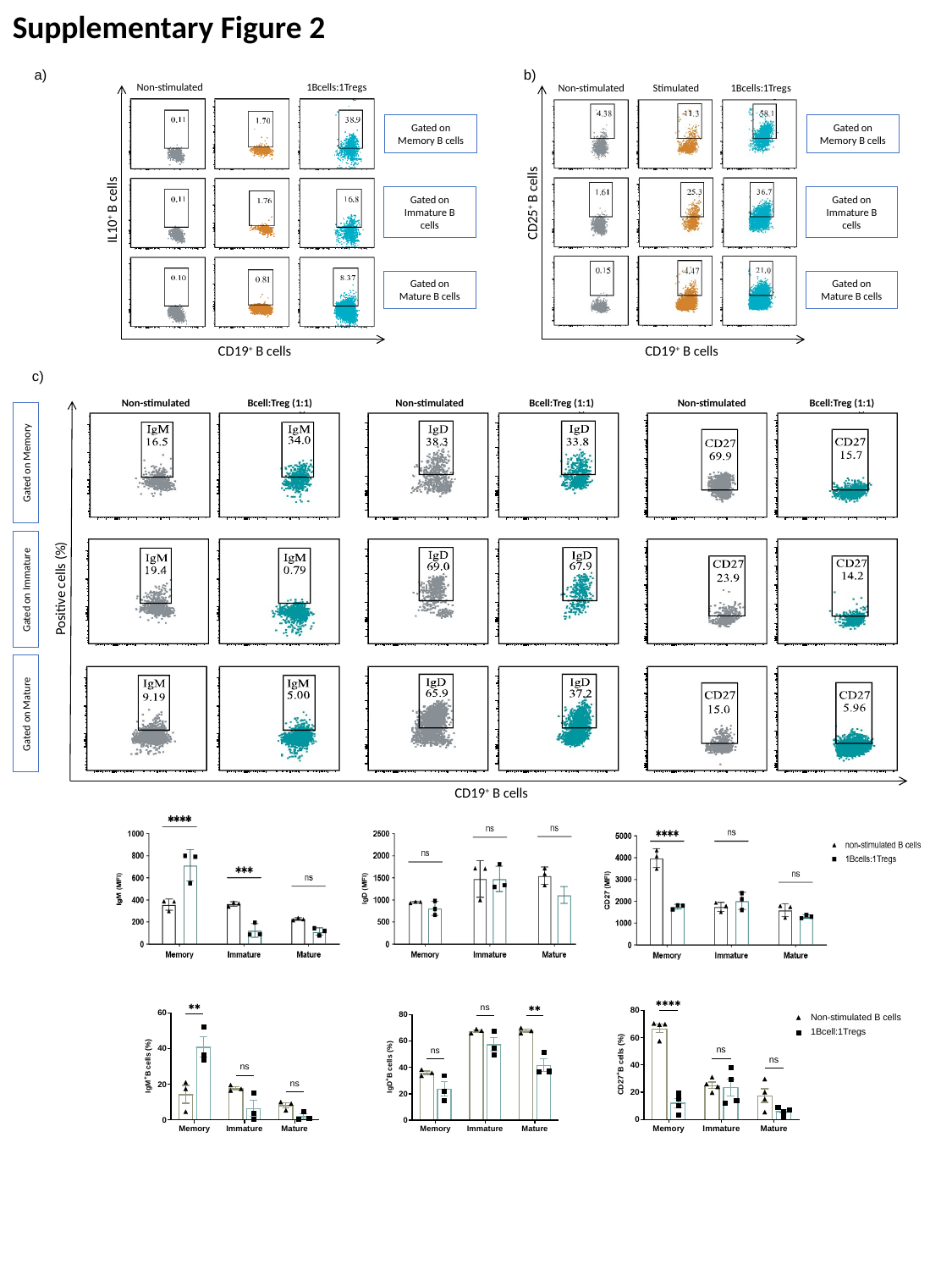

Supplementary Figure 2
a)
b)
Non-stimulated
1Bcells:1Tregs
Non-stimulated
Stimulated
1Bcells:1Tregs
Gated on Memory B cells
Gated on Memory B cells
Gated on Immature B cells
Gated on Immature B cells
CD25+ B cells
IL10+ B cells
Gated on Mature B cells
Gated on Mature B cells
CD19+ B cells
CD19+ B cells
c)
Non-stimulated
Bcell:Treg (1:1)
Non-stimulated
Bcell:Treg (1:1)
Non-stimulated
Bcell:Treg (1:1)
Gated on Memory
Positive cells (%)
Gated on Immature
Gated on Mature
CD19+ B cells

## Slide 8
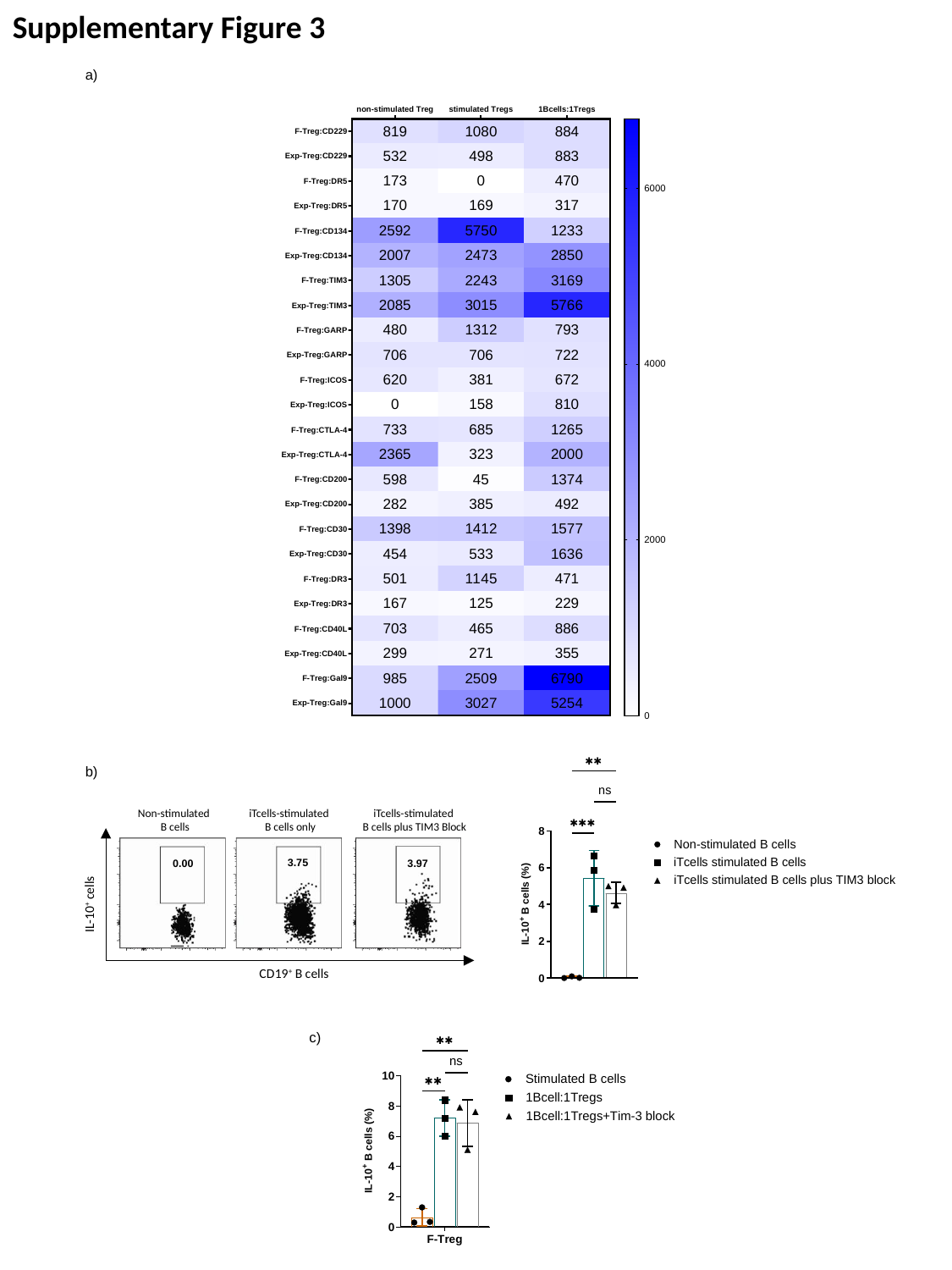

Supplementary Figure 3
a)
b)
Non-stimulated
 B cells
iTcells-stimulated
 B cells only
iTcells-stimulated
 B cells plus TIM3 Block
3.75
0.00
3.97
IL-10+ cells
CD19+ B cells
c)

## Slide 9
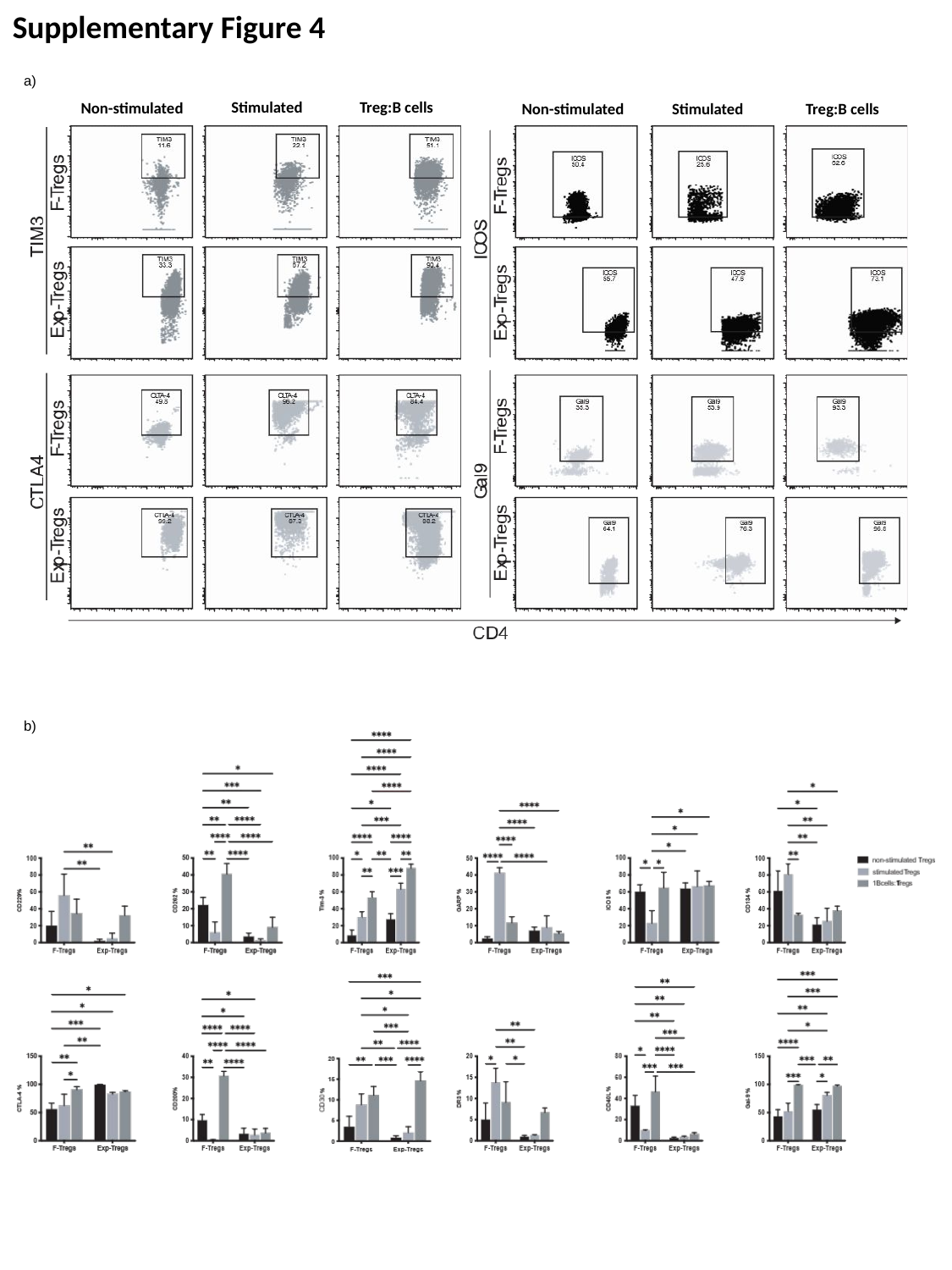

Supplementary Figure 4
a)
Stimulated
Treg:B cells
Non-stimulated
Non-stimulated
Stimulated
Treg:B cells
b)
